# CD248 facilitates tumor growth via its cytoplasmic domain

**DOI:** 10.1186/1471-2407-11-162

**Published:** 2011-05-08

**Authors:** Margarida Maia, Astrid DeVriese, Tom Janssens, Michaël Moons, Rik J Lories, Jan Tavernier, Edward M Conway

**Affiliations:** 1 Vesalius Research Center, VIB, Herestraat 49, 9th floor, 3000 Belgium; 2 Vesalius Research Center, K. U. Leuven, Herestraat 49, 9th floor, 3000 Belgium; 3 Cytokine Receptor Laboratory, Department of Medical Protein Research, VIB, A. Baertsoenkaai 3, 9000 Ghent, Belgium; 4 Cytokine Receptor Laboratory, Department of Biochemistry, Ghent University, A. Baertsoenkaai 3, 9000 Ghent, Belgium; 5 Laboratory for Skeletal Development and Joint Disorders, Division of Rheumatology, K. U. Leuven, Herestraat 49, 3000 Belgium; 6 Centre for Blood Research, Faculty of Medicine, Division of Hematology, University of British Columbia, 2350 Health Sciences Mall Vancouver, V6T 1Z3, Canada

**Keywords:** stromal fibroblast, suppressor, transgenic, endosialin, tumor endothelial marker

## Abstract

**Background:**

Stromal fibroblasts participate in the development of a permissive environment for tumor growth, yet molecular pathways to therapeutically target fibroblasts are poorly defined. CD248, also known as endosialin or tumor endothelial marker 1 (TEM1), is a transmembrane glycoprotein expressed on activated fibroblasts. We recently showed that the cytoplasmic domain of CD248 is important in facilitating an inflammatory response in a mouse model of arthritis. Others have reported that *CD248 *gene inactivation in mice results in dampened tumor growth. We hypothesized that the conserved cytoplasmic domain of CD248 is important in regulating tumor growth.

**Methods:**

Mice lacking the cytoplasmic domain of CD248 (CD248^CyD/CyD^) were generated and evaluated in tumor models, comparing the findings with wild-type mice (CD248^WT/WT^).

**Results:**

As compared to the response in CD248^WT/WT ^mice, growth of T241 fibrosarcomas and Lewis lung carcinomas was significantly reduced in CD248^CyD/CyD ^mice. Tumor size was similar to that seen with CD248-deficient mice. Conditioned media from CD248^CyD/CyD ^fibroblasts were less effective at supporting T241 fibrosarcoma cell survival. In addition to our previous observation of reduced release of activated matrix metalloproteinase (MMP)-9, CD248^CyD/CyD ^fibroblasts also had impaired PDGF-BB-induced migration and expressed higher transcripts of tumor suppressor factors, transgelin (SM22α), Hes and Hey1.

**Conclusions:**

The multiple pathways regulated by the cytoplasmic domain of CD248 highlight its potential as a therapeutic target to treat cancer.

## Background

In normal tissues, fibroblasts are the major cellular component of connective tissue and are key participants in maintaining homeostasis of the extracellular matrix (ECM), regulating epithelial differentiation, inflammation and wound healing. Fibroblasts not only synthesize the major constituents of the ECM, but they release ECM-degrading proteinases to assure normal matrix turnover and function. Fibroblasts also secrete multiple growth factors and support mesenchymal-epithelial interactions via paracrine and juxtacrine signaling. Within the tumor stroma, subpopulations of fibroblasts emerge and exhibit an "activated" phenotype, whereupon they acquire characteristics that can be distin guished from normal fibroblasts and often portend a bad prognosis [1]. These activated fibroblasts, also referred to as peritumoral fibroblasts, cancer-associated fibroblasts, reactive stromal fibroblasts and tumor-associated fibroblasts, are characterized by the expression of myofibroblast-like cell markers, including alpha smooth muscle actin (α-SMA) and desmin, and secrete factors that generally promote cell growth and proliferation (e.g. hepatocyte growth factor (HGF), epidermal growth factor (EGF), vascular endothelial growth factor (VEGF), insulin growth factor 2 (IGF2), fibroblast-like growth factor 2 (FGF2), transforming growth factor-beta (TGF-β), ECM-degrading proteinases such as MMPs, cytokines such as tumor necrosis factor (TNF)-αand interleukin (IL)-1β and chemokines [2-4].

Tumor-associated fibroblasts are believed to originate from tissue-resident fibroblasts and mesenchymal stem cells, by recruitment of bone marrow-derived cells from the circulation [5] and/or by epithelial-to-mesenchymal transition [6]. The mechanisms by which fibroblasts become activated are not well-defined, although TGF-β, EGF, platelet-derived growth factor (PDGF)-BB, FGF2, reactive oxygen species, complement factors, and integrins have all been implicated [7-9]. Although there are major gaps in our understanding of the mechanisms by which tumor-associated fibroblasts evolve, cell surface markers that are specific to these cells are attractive candidate targets for therapy.

CD248, also referred to as endosialin or tumor endothelial marker 1 (TEM1), is a highly sialylated cell surface glycoprotein [10-12] that has been shown to be restricted to activated stromal and perivascular fibroblasts [13-16]}. During normal embryonic development, CD248 is highly expressed [17,18], but by full-term, CD248 has almost entirely disappeared. Postnatally, expression is retained only in the endometrium, in bone marrow fibroblasts and in the corpus luteum [11,15,19]. However, CD248 is frequently upregulated in tumors [10,15,20], with particularly high expression in tumor associated stromal fibroblasts in sarcomas [19] and primary and secondary brain tumors [21]. CD248 is also expressed in human mesenchymal stem cells from bone marrow, which may differentiate into tumor stromal fibroblasts [22]. In breast cancer and neuroblastomas, CD248 expression levels have been directly correlated with tumor grade, invasiveness and poor prognosis [23,24]. The physiologic importance of CD248 in cancer progression and its potential utility as a therapeutic target is further highlighted by the finding that lack of CD248 in mice results in resistance to the growth and metastasis of some tumors [25]. Therefore, delineating the hitherto unknown mechanisms by which CD248 regulates tumor growth is important for the development of therapeutic strategies.

The human *CD248 *gene is intronless and encodes a 95-kDa multi-domain type I transmembrane protein of 757 amino acids [26]. The protein comprises an N-terminal C-type lectin-like domain, a Sushi domain, three epidermal growth factor (EGF)-like repeats, a mucin-like region, a single transmembrane segment and a 51 amino acid residue cytoplasmic tail with potential sites for phosphorylation [27]. CD248 belongs to a family of proteins containing C-type lectin-like domains which have functions in cell adhesion and regulation of inflammation [28,29].

Few analyses have been performed to elucidate the mechanisms by which CD248 regulates tumor growth. *In vitro *studies suggest that the extracellular region of CD248 may interact with ECM proteins, thereby facilitating activation of MMP-9, cell migration and metastasis formation [30,31]. We recently demonstrated that the highly conserved cytoplasmic domain of CD248 mediates signals that regulate stromal fibroblast function in an experimental model of rheumatoid arthritis [32] and hypothesized that it would play a similarly important role in modulating tumor growth. We show that lack of the cytoplasmic domain of CD248 in transgenic mice results in reduced tumor growth, with alterations in fibroblast signaling via TGF-β, PDGF-BB, and Notch pathways, and establishment of a pattern of gene expression favoring tumor suppression. The findings extend previous reports of the importance of CD248 in tumor growth and point to the cytoplasmic domain of CD248 as a potential therapeutic target in neoplasia.

## Methods

### Mice

Transgenic mice lacking CD248 (CD248^KO/KO^) or the cytoplasmic domain of CD248 (CD248^CyD/CyD^) were previously generated and genotyped as reported [32]. Mice were maintained on a C57Bl6 genetic background and corresponding wild-type mice (CD248^WT/WT^), generated from siblings during breeding of the CD248 transgenic lines, were used as controls.

### In vivo tumor models

Heterotopic implantation of Lewis Lung Carcinoma (LLC) tumor fragments was performed as described in [25]. Briefly, 0.5 × 10^6 ^LLC cells were injected subcutaneously (s.c.) into the right flank of 5-week-old CD248^WT/WT ^mice. After 20 days, mice were sacrificed and tumors dissected and cut into 1 mm^3 ^pieces. 6-7 week old mice were anaesthetized with isoflurane and the cecum exteriorized via a small incision parallel to the midline. A single tumor fragment was implanted on the serosal surface of the cecum. Tumors were dissected 15 days after implantation, and tumor volume and weight were measured. Volume was calculated using the formula, length × width^2 ^× π/6.

For T241 fibrosarcoma studies, 1 × 10^6 ^cells in 200 μL PBS or 7.5 × 10^4 ^LLC cells in 50 μL PBS were injected s.c. into the right flank or footpad of 7-9 week-old mice. Tumor size was evaluated every 2 days using a caliper and weights were obtained after dissection. Studies were performed in a blinded manner to the investigator.

The model of orthotopic growth and metastasis of pancreatic adenocarcinoma in mice was performed exactly as reported [33]. Briefly, mice were anaesthetized with isoflurane and the stomach exteriorized via an abdominal midline incision. 1 × 10^6 ^PancO2 pancreatic adenocarcinoma cells in 25 μL PBS were injected into the head of the pancreas. At day 11, primary tumors were dissected, and tumor volume and weight were determined.

### Immunohistochemistry and quantification of tumor vessel density

Tissue samples were fixed with 2% paraformaldehyde overnight at 4°C, dehydrated and embedded in paraffin. Serial 7 μm sections were cut for histological analysis. Immunohistochemical detection was performed using the following antibodies: rat anti-CD31 (BD Pharmingen, Erembodegem, Belgium), mouse anti-SMA-Cy3 (Dako, Glostrup, Denmark), phospho-histone H3 (Cell Signaling Technology, Bioké, Leiden, the Netherlands) and rabbit anti-caspase 3 (Abcam, Cambridge, MA, USA). Morphometric analyses were performed using a Zeiss Imager Z1 or AxioPlan 2 microscope with KS300 image analysis software. For all studies, 6-8 optical fields per tumor section, at 40× or 80× magnification, were randomly chosen and analyzed. Cell proliferation was calculated as the number of phospho-histone H3 per mm^2^. Vessel density was calculated as the number of CD31-positive vessels per mm^2 ^and pericyte coverage as the percentage of CD31-positive vessels that are covered by SMA-positive cells. Vessel distribution was determined by calculating the frequency distribution of vessels with different areas.

### In vitro studies with murine embryonic fibroblasts

Murine embryonic fibroblasts were isolated from E13.5 embryos as previously reported [32]. Fibroblasts were cultured in DMEM with 10% fetal calf serum (FCS) and used at passages 2-5. To assess the response to exogenous growth factors, 1.5 × 10^5 ^fibroblasts were seeded in 6-well plates. After 18 hours of serum-starvation, cells were stimulated with 20 ng/mL recombinant rat PDGF-BB (R&D Systems, Abingdon, UK) for 30 minutes or 3 ng/mL recombinant human TGF-β1 (R&D Systems) for 72 hours. To assess the role of direct contact of endothelial cells, 1.5 × 10^5 ^fibroblasts were mixed with an equal number of the human endothelial cell line, EaHy926 [34] and cultured in DMEM containing 10% FCS for 24 hours. Cells were finally processed for reverse transcription PCR.

### Murine embryonic fibroblast migration studies

2.5 × 10^4 ^fibroblasts were seeded in the upper chamber of 8 μm-pore size transwells (Costar, Elscolab, Kruibeke, Belgium). DMEM/1% FBS with or without 20 ng/mL PDGF-BB was added to the bottom well to stimulate migration. After 18 hours of incubation, cells were fixed in 1% paraformaldehyde and stained with 0.5% crystal violet solution. The number of migrated cells was quantified by counting five high-power magnification fields per transwell [14]. All studies were repeated with 3 independent clones of fibroblasts from each genotype, yielding comparable results. Thus, representative results were reported.

### Tumor cell survival assays

5 × 10^3 ^T241 fibrosarcoma cells or PancO2 cells were seeded in 96-well plates and serum-starved for 6 hours before adding conditioned medium of murine embryonic fibroblasts grown in DMEM containing 10% FCS or stimulated with serum-free DMEM containing 3 ng/mL recombinant human TGF-β1 for 24 hours. The number of viable cells was determined 24, 48 and 72 hours after using the CellTiter 96 Aqueous One Solution (Promega, Leiden, The Netherlands) according to manufacturer's instructions.

### Quantitative reverse transcription (qRT)-PCR

RNA was extracted from cells using Qiagen RNeasy kit. 0.5-1 μg of total RNA was used for reverse transcription with QuantiTect Reverse Transcription kit (Qiagen, KJ Venlo, the Netherlands). qRT-PCR was performed using TaqMan Fast Universal PCR Master Mix (Applied Biosystems, Halle, Belgium) and commercially available or home-made primers and probes for the genes of interest (Table 1). Analyses were performed using ABI7500 Fast Real-Time PCR System (Applied Biosystems, Halle, Belgium). All experiments were performed a minimum of 3 times, each in at least triplicate.

**Table 1:** List of primers and probes used for qRT-PCR

Gene	Forward	Reverse	Probe
Hes	TCAGCGAGTGCATGAACGA	CCTCGGTGTTAACGCCCTC	TGACCCGCTTCCTGTCCACGTG

**Gene**	**Sequence ID (Commercially available primers)**		

β-actin	Mm00607939_s1		

CD248	Mm0054785_s1		

Hey1	Mm00468865_m1		

Jagged1	Mm00496902_m1		

hJagged1	Hs01070028_g1		

Notch3	Mm00435270_m1		

SM22α	Mm00441660_m1		

### Statistics

Data represent mean ± standard error of the mean (SEM) of experiments performed at least in duplicate. Statistical significance was calculated by t-test or two-way ANOVA (Prism 5.0), with p < 0.05 considered statistically significant.

### Animal care

All experimental animal procedures were approved by the Institutional Animal Care and Research Advisory Committee of the K. U. Leuven.

## Results

### The cytoplasmic domain of CD248 is required for the growth of some tumors

**Figure 1:**
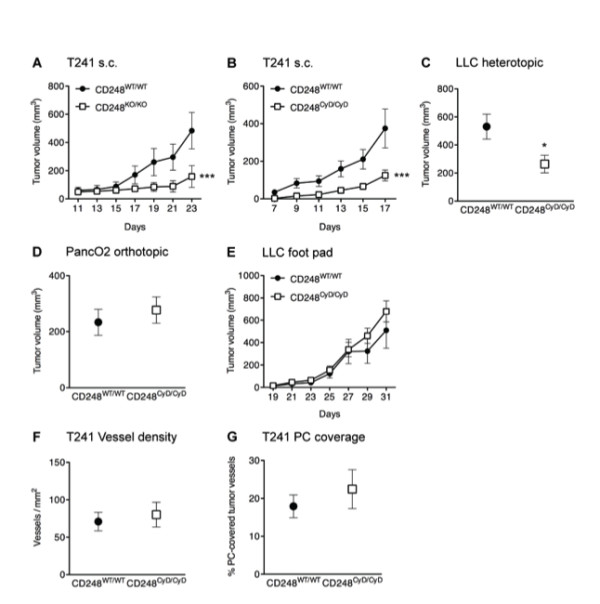
**Role of CD248 in tumor growth in mice**. **A**. Growth of T241 tumors is significantly reduced in CD248^KO/KO ^mice (n = 6, p < 0.001). **B**. Growth of T241 tumors is significantly reduced in CD248^CyD/CyD ^mice (n = 6, p < 0.001). **C**. LLC tumors implanted in the cecum are significantly smaller in CD248^CyD/CyD ^mice (n = 7, p < 0.05). **D**. PancO2 tumors grown orthotopically are not different in size in CD248^WT/WT ^and CD248^CyD/CyD ^mice (n = 4, p = not significant). **E**. LLC tumor growth in the footpad is similar in CD248^WT/WT ^and CD248^CyD/CyD ^mice (n = 6, p = not significant). **F-G**. Morphometric quantification reveals that there are no genotype-dependent differences in vessel density (F) or PC coverage (G) (n = 7, p = not significant). Data represent the mean ± SEM. Asterisks denote statistical significance.

Studies with CD248-deficient mice indicate that CD248 is necessary for tumor growth [25]. In an experimental model of rheumatoid arthritis, we recently determined that CD248^KO/KO ^mice and mice lacking the cytoplasmic domain of CD248 (CD248^CyD/CyD ^mice) develop less severe arthritis than CD248^WT/WT ^mice, the differential response likely due to alterations in synovial fibroblast function [32]. Based on these data, we predicted that the highly conserved cytoplasmic domain of CD248 would mediate signals that critically contribute to tumor growth. To test this hypothesis, we first validated the role of CD248 in tumor growth in CD248^KO/KO ^mice, comparing the response with corresponding CD248^WT/WT ^mice. T241 fibrosarcoma cells were injected s.c. and tumor size was monitored. Over a period of 23 days, tumors in the CD248^KO/KO ^mice were significantly smaller than in the CD248^WT/WT ^mice (n = 6, p = 0.0009) (Figure 1A). The findings are confirmatory of an important role for CD248 in tumor growth.

We next assessed whether the cytoplasmic domain of CD248 is required for growth of the T241 fibrosarcomas. We observed a significant reduction in tumor size and tumor weight of s.c. T241 fibrosarcoma tumors in the CD248^CyD/CyD ^mice as compared to CD248^WT/WT ^mice (n = 6, p < 0.0001) (Figure 1B). In experiments replicating the setup of Nanda and coworkers [25], we also implanted LLC tumor fragments heterotopically on the serosal surface of the cecum of CD248^WT/WT ^or CD248^CyD/CyD ^mice. Again, CD248^CyD/CyD ^mice developed tumors that were significantly smaller than in the CD248^WT/WT ^mice (n = 7, p < 0.05) (Figure 1C).

Notably, not all tumors were resistant to growth in the CD248^CyD/CyD ^mice, i.e. there were no differences in the growth of primary orthotopic PancO2 pancreatic adenocarcinomas (Figure 1D) or after injection of LLC cells into the footpads (Figure 1E). Thus, similar to what was observed by others with CD248-deficient mice [25], CD248 does not regulate the growth of all tumors.

### Angiogenesis-independent reduced tumor growth in CD248^CyD/CyD ^mice

Although the dependence of tumor progression on angiogenesis is well-documented [35], additional factors also modify tumor growth, sometimes apparently independent of new vessel growth [36,37]. This is relevant to CD248, as two groups have reported a CD248-dependent inverse relationship between microvessel density and size of tumor. Tumors of the large intestine derived from LLC cells [25] and intracranial glioblastomas derived from U87MG cells [21] were reportedly smaller in size and weight in CD248-deficient mice, yet these tumors had higher vessel density than those in wild-type mice. We assessed the role of the cytoplasmic tail of CD248 in modulating the relationship between tumor growth and angiogenesis by first examining vessel density in the s.c. T241 fibrosarcomas in CD248^CyD/CyD ^and CD248^WT/WT ^mice at the end of the observation period. Endothelial cell-specific CD31 staining of the tumors revealed that vessel density was not significantly different in tumors from CD248^WT/WT ^and CD248^CyD/CyD ^mice (Figure 1F). We also did not detect differences based on genotype when we stratified the results according to the lumen area of the vessels (data not shown). The number of vessels covered by SMA-positive pericytes was also not affected by lack of the cytoplasmic domain of CD248 (Figure 1G). Notably, we did not observe an *inverse *relationship between vessel density and tumor size in our studies with the CD248^CyD/CyD ^*versus *CD248^WT/WT ^mice, findings that contrast with those reported in studies using CD248-deficient mice [21,25]. This prompted us to examine T241 tumor angiogenesis in our CD248^KO/KO ^mice, with the aim of establishing whether the cytoplasmic domain is the major determining factor. In spite of significantly smaller tumors in the CD248^KO/KO ^*versus *CD248^WT/WT ^mice at the time of sacrifice at Day 23 (Figure 1A), vessel density and pericyte coverage of s.c. T241 fibrosarcomas in CD248^WT/WT ^and CD248^KO/KO ^mice were not significantly different (endothelial-specific CD31 staining, vessels per mm^2^: 107 ± 18 vessels/mm^2 ^in CD248^WT/WT ^*versus *90 ± 8 vessels/mm^2 ^in CD248^KO/KO^; n = 7, p = 0.40; % vessels covered by smooth muscle actin (SMA)-positive cells: 23.6 ± 5.3 in CD248^WT/WT ^*versus *26.7 ± 3.1 in CD248^KO/KO^; n = 7, p = 0.619).

Overall, the preceding findings indicate that loss of CD248 or its cytoplasmic domain results in an uncoupling of the link between tumor growth and vessel density, and that alterations in stroma-derived factors regulated by CD248 may underlie the differences in tumor growth.

### Cytoplasmic domain of CD248 modulates stromal fibroblast function

Using murine embryonic fibroblasts as a model [38-40], we recently established that in spite of having normal antigen levels, fibroblasts from CD248^CyD/CyD ^mice are less adhesive to monocytoid cells and express lower levels of placental growth factor (PlGF), VEGF, VEGF receptor-1 (VEGFR-1) and matrix metalloproteinase (MMP)-9 [32]. These findings are consistent with our observation that the CD248^CyD/CyD ^mice are resistant to tumor growth, but suggest that there may be multiple pathways by which the cytoplasmic domain of CD248 regulates fibroblast function to facilitate tumor progression. We therefore tried to uncover additional CD248-dependent alterations in stromal fibroblasts that could help to explain the smaller tumors in CD248^CyD/CyD ^mice.

### a. Elevated expression of the tumor suppressor SM22α in CD248^CyD/CyD ^fibroblasts

**Figure 2:**
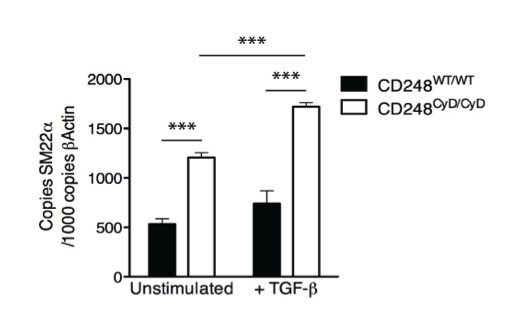
**CD248-dependent expression of SM22α**. CD248^CyD/CyD ^fibroblasts express higher transcript levels of SM22α at baseline (n = 4, p < 0.0001) and after TGF-β stimulation for 72 hours (n = 4, p = 0.0004). Results reflect the mean ± SEM. Asterisks denote statistical significance.

Recent studies support the concept that activated stromal fibroblasts co-evolve with tumor cells and that their activation and survival is sustained in part by altered expression of tumor suppressor genes [41,42]. Transgelin (SM22α) is a cytoplasmic actin-binding protein, expressed by mesenchymal cells and tumor fibroblasts [43] and which represses TGF-β-induced MMP-9 expression [44], reduces cell migration, and has tumor suppressor properties [45]. We assessed whether expression of SM22α is dependent on the integrity of the cytoplasmic domain of CD248. Baseline transcript levels of SM22α were significantly higher in CD248^CyD/CyD ^fibroblasts as compared to CD248^WT/WT ^fibroblasts (Figure 2). Transcript levels of SM22α in the CD248^WT/WT ^fibroblasts increased to a non-significant extent in response to TGF-β. In contrast, the transcript levels of SM22α, already elevated in the CD248^CyD/CyD ^fibroblasts, increased further upon TGF-β stimulation to levels significantly higher than in the TGF-β stimulated CD248^WT/WT ^fibroblasts. The findings indicate that the cytoplasmic domain of CD248 participates in the suppression of SM22α expression, providing a mechanism to help explain reduced stromal cell activation, migration and MMP-9 release and thus, less tumor expansion in the CD248^CyD/CyD ^mice.

### b. The cytoplasmic domain of CD248 facilitates PDGF-BB-induced cell migration

**Figure 3:**
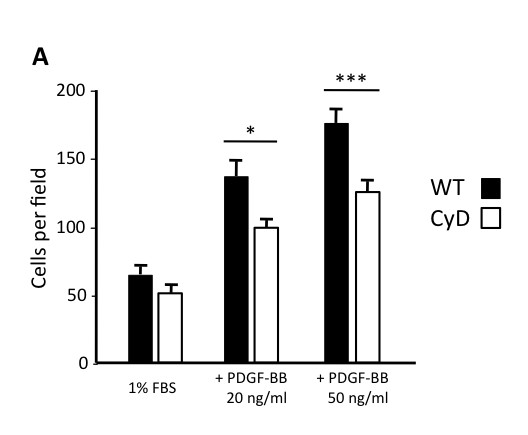
**CD248 modulates PDGF-BB mediated cell migration**. **A**. Migration of fibroblasts across a transwell was quanitified as described in Methods. PDGF-induced migration of CD248^CyD/CyD ^fibroblasts is significantly reduced as compared with CD248^WT/WT ^fibroblasts (n = 6). Results reflect the mean ± SEM. * p < 0.05; ** p < 0.001.

With altered expression of SM22α, we hypothesized that CD248^CyD/CyD ^fibroblasts would also exhibit resistance to factors that promote their migration in a tumor setting. PDGF-BB is a potent chemoattractant for stromal cells and a trigger for recruitment of tumor associated fibroblasts (reviewed in [46]). Experimental evidence supports a role for PDGF signaling in cancer progression, with rationale for targeting this pathway in tumor-associated stromal fibroblasts [47,48]. Moreover, it has been shown in some studies that fibroblasts require CD248 for optimal migratory response [14,49]. To determine whether the cytoplasmic domain of CD248 regulates PDGF-BB-induced cell migration, CD248^WT/WT ^and CD248^CyD/CyD ^fibroblasts were stimulated to migrate across transwells toward recombinant PDGF-BB. In the absence of PDGF-BB, there were no differences in the number of migrated CD248^WT/WT ^and CD248^CyD/CyD ^fibroblasts. For both genotypes, PDGF-BB induced a significant increase in fibroblast migration in a dose-dependent manner. However, at the two concentrations of PDGF-BB tested, CD248^CyD/CyD ^fibroblast migration was significantly less as compared to the migration of CD248^WT/WT ^fibroblasts (Figure 3). Overall, these data support the importance of the cytoplasmic domain of CD248 in facilitating PDGF-BB mediated stromal fibroblast migration, a process that is important in tumorigenesis.

### c. CD248 suppresses Notch3 signaling and dampens Hes and Hey1 gene expression

Although activation of Notch may promote cancer development [50], Notch may also have a tumor suppressor function, partly by promoting cellular differentiation and maturation of mesenchyme derived perivascular cells [51,52]. Notch signaling requires activation by the ligand Jagged1, which induces cleavage of Notch3 and release of Notch intracellular domain (NICD), nuclear translocation of which further activates Notch3. This is accompanied by transcription of several genes, including Hes, Hey1 and SM22α, all of which may exhibit tumor suppressor properties [45,53,54]. Notch3 also feeds back and promotes Jagged1 expression in pericytes, thereby maintaining their differentiated state [55].

**Figure 4:**
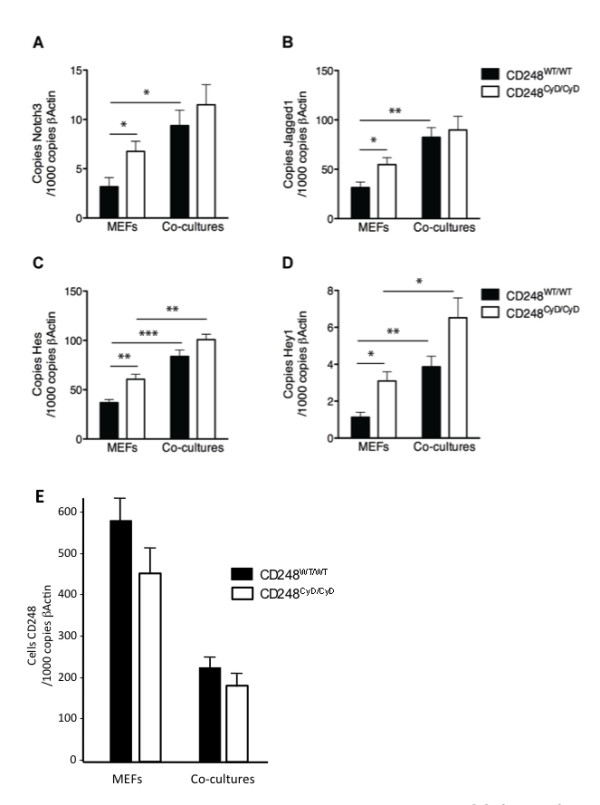
**Effects of CD248 on Notch3 signaling**. Baseline transcript levels of Notch3 (**A**), Jagged1 (**B**), Hes (**C**) and Hey1(**D**), are increased in CD248^CyD/CyD ^fibroblasts as compared with CD248^WT/WT ^fibroblasts(n = 4, p = 0.0139 for Notch3; p = 0.0041 for Jagged1; p = 0.00067 for Hes; p = 0.0051 for Hey1). Fibroblasts were also co-cultured with human EaHy926 endothelial cells for 24 hours (**A-D**), causing further upregulation of the transcripts for both genotypes. **E**. Transcript levels of CD248 in CD248^WT/WT ^and CD248^CyD/CyD ^fibroblasts are significantly reduced to similar levels after co-culture with EaHy926 endothelial cells (n = 4).

To determine whether the cytoplasmic domain of CD248 alters Notch3 signaling, we measured genes involved in the Notch pathway in isolated fibroblasts and in fibroblasts that were co-cultured with equal numbers of human EaHy926 endothelial cells. Baseline levels of Notch3, Jagged1, Hes and Hey1 transcripts were significantly increased in CD248^CyD/CyD ^fibroblasts as compared with CD248^WT/WT ^fibroblasts (Figure 4), indicating that Notch3 signaling and transcription of Hes and Hey1 in isolated fibroblasts is dampened via signals mediated by the cytoplasmic domain of CD248.

Human endothelial Jagged1 is known to induce Notch signaling in murine fibroblasts [55] and the use of different species cells in co-culture allowed us to distinguish the cellular source of each gene. As expected, contact of endothelial cells with CD248^WT/WT ^fibroblasts resulted in an increase in expression of the transcripts for Notch3, Jagged1, Hes and Hey1 (Figure 4). When CD248^CyD/CyD ^fibroblasts were co-cultured with endothelial cells, transcript levels of Notch3, Jagged1, Hes and Hey1 also increased as compared with fibroblasts alone. Responses were not affected by changes in endothelial expression of Jagged1, since Jagged1 was similarly upregulated in endothelial cells co-cultured with either CD248^CyD/CyD ^or CD248^WT/WT ^fibroblasts (copies of human Jagged1 per 1000 copies of β-actin: 740 + 70 in co-cultures with CD248^WT/WT ^fibroblasts *versus *710 + 70 in co-cultures with CD248^CyD/CyD ^fibroblasts, n = 4, p = not significant). Although Notch3, Hes and Hey1 gene expression were still higher in CD248^CyD/CyD ^fibroblast than in CD248^WT/WT ^fibroblasts in the co-culture conditions, the differences did not achieve statistical significance. We considered that this might be an *in vitro *effect due to endothelial contact-induced differentiation and maturation of the fibroblasts, which would thus suppress CD248 expression and diminish the differential effect of the absent cytoplasmic domain of the molecule on Notch signaling. Indeed, under these co-culture conditions, CD248 transcript levels were significantly reduced by >50% in both the CD248^WT/WT ^and CD248^CyD/CyD ^fibroblasts (Figure 4E).

Taken together, the findings support the concept that suppression of CD248 or interfering with signaling via its cytoplasmic domain may be associated with increased maturation of tumor stromal or perivascular fibroblasts and expression of tumor suppressor genes.

### CD248-dependent release of soluble factors from fibroblasts modulates T241 tumor cell proliferation

**Figure 5:**
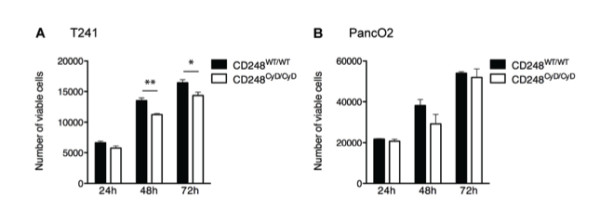
**CD248-dependent fibroblast release of trophic factors that regulate tumor cell survival**. T241 fibrosarcoma cells (**A**) and PancO2 pancreatic adenocarcinoma cells (**B**) were grown in conditioned media (CM) from CD248^WT/WT ^fibroblasts and CD248^CyD/CyD ^fibroblasts and cell viability was quantified at 24, 48 and 72 hours as described in Methods. At 48 hours (n = 3) and 72 hours (n = 6), the number of T241 cells exposed to CM from the CD248^CyD/CyD ^fibroblasts was significantly lower as compared to those exposed to CM from the CD248^WT/WT ^fibroblasts. No differences in viability were detected with the PancO2 cells. Data represent the mean ± SEM. * p < 0.05; ** p < 0.01.

The preceding studies underscore the role of the cytoplasmic domain of CD248 in modulating genes that regulate stromal fibroblast differentiation, maturation and migration and the expression of tumor suppressor genes. We recently reported that CD248^CyD/CyD ^fibroblasts express reduced amounts of VEGF, PlGF and active MMP-9 [32] and considered that other soluble factors might be secreted in a CD248-dependent manner that regulate tumor cell proliferation. We therefore assessed the effects of conditioned media (CM) from the fibroblasts of CD248^WT/WT ^and CD248^CyD/CyD ^on T241 fibrosarcoma cell proliferation. After 24 hours, tumor cell viability with CM from the different genotype fibroblasts was not significantly different (Figure 5A). However, at 48 hours and 72 hours, the number of T241 fibrosarcoma cells exposed to CM from the CD248^CyD/CyD ^fibroblasts was significantly lower as compared to those exposed to CM from the CD248^WT/WT ^fibroblasts. Although there appeared to be a similar CD248-dependent effect on PancO2 cell survival, the differences did not achieve statistical significance (Figure 5B). These findings, which are consistent with those observed *in vivo*, indicate that the cytoplasmic domain of CD248 modulates the release of factors from fibroblasts that differentially promote the survival of specific tumor cells.

## Discussion

CD248 was originally believed to be a tumor endothelial cell marker, and thus referred to as endosialin or TEM1 [10-12]. CD248 is now recognized to be primarily expressed on the surface of mesenchymal stem cells, activated stromal fibroblasts and pericytes [16], cells that may contribute to fibrovascular network expansion and tumor progression [5]. While several investigators have shown that CD248 plays an important role in tumor growth and stromal expansion [13-15,21,25] with expression levels that have been correlated with tumor progression [23,24], the mechanisms by which CD248 functions and the key structural domains involved, have remained a mystery. In our studies, we established that the cytoplasmic domain of CD248 is a key regulator of tumor growth and that mice lacking this domain are resistant to growth of T241 fibrosarcoma tumors and heterotopic LLC tumors.

Activated fibroblasts dynamically interact with constituents of the stromal compartment and participate in tumor progression via several mechanisms, including for example, remodeling the ECM, promoting the recruitment of inflammatory cells, enhancing nutritional support of the stromal microenvironment, and by secreting an array of pro-lymph/angiogenic and autocrine and paracrine acting cellular growth factors [6,56-58]. The cytoplasmic domain of CD248 is a critical enabler for stromal fibroblast activation, endowing the fibroblast with several tumor-promoting properties. Tumor fibroblasts can achieve and maintain an activated state in the tumor microenvironment by acquiring epigenetic and/or genetic changes that mitigate the function of tumor suppressor genes, such as p53 and PTEN in breast cancer [41,42]. In this manner, activated fibroblasts can exhibit a phenotype that favors proliferation and an enhanced response to pro-survival and migratory cues released by neighboring fibroblasts and other tumor and non-tumor stromal cells. In this regard, we evaluated the relationship between the cytoplasmic domain of CD248 and SM22α, a tumor suppressor gene that when dysregulated, is implicated in the progression and metastasis of cancers of the colon, breast and prostate [45,59]. SM22α was considered a likely candidate for CD248-dependent expression because it is known to be upregulated by TGF-β, and because we had previously shown that CD248^CyD/CyD ^fibroblasts release less TGF-β and are resistant to TGF-β-mediated angiogenic and pro-inflammatory activities [32]. CD248^CyD/CyD ^fibroblasts expressed significantly higher transcript levels of SM22α, further upregulated by TGF-β to levels exceeding those seen with CD248^WT/WT ^fibroblasts. Although we have not directly determined whether SM22α dampens activation of fibroblasts, it is reasonable to consider that elevated levels might contribute to the smaller tumors in the CD248^CyD/CyD ^mice by maintaining the fibroblasts in a more quiescent state and by indirectly reducing TGF-β-induced MMP-9 release, overall preventing microenvironmental changes that would facilitate cell migration and cancer progression.

Since SM22α is co-ordinately regulated by Notch and TGF-β [60], we hypothesized that the cytoplasmic domain of CD248 would similarly regulate expression of Hes and Hey1, downstream effectors of Notch that also exhibit context-specific tumor suppressor properties [53,54]. Indeed, Hes and Hey1 transcript levels in CD248^CyD/CyD ^fibroblasts were significantly higher than in CD248^WT/WT ^fibroblasts, in line with the role of CD248 in promoting tumor growth. Notably from these studies, we uncovered a novel mechanism by which fibroblast CD248 is itself regulated, i.e., it is markedly suppressed by direct contact with endothelial cells. Although the molecular mechanisms remain to be clarified, identification of strategies to downregulate CD248 will be important for the design of therapies to reduce both tumor growth and inflammation.

In addition to the role of the cytoplasmic domain of CD248 in imparting fibroblast sensitivity to the effects of TGF-βα, our studies also show that this domain of CD248 is crucial for optimal migratory response of activated fibroblasts to PDGF-BB. Our observations are in line with recent reports showing that CD248-deficient fibroblasts or pericytes also have defects in migration and proliferation that may [14,61] or may not [49] depend on PDGF-BB. The apparent discordant findings in the literature likely reflect differences in experimental approaches. Studies by Tomkowicz et al. suggest that CD248 may recruit Src/PI-3 Kinase and cFos pathways to enhance PDGF-BB-induced signals emanating from the PDGF-receptor [61]. Further study will be necessary to elucidate the intracellular pathways responsible for the reduced PDGF-BB induced CD248^CyD/CyD ^fibroblast migration.

In exploring the mechanisms underlying increased resistance to arthritis induction in CD248^CyD/CyD ^mice [32], we recently showed that CD248^CyD/CyD ^fibroblasts are less adherent to monocytes and express reduced levels of VEGF, PlGF and VEGFR-1. These CD248-dependent alterations serve to reduce leukocyte infiltration, synovial fibroblast migration, proliferation and inflammation in arthritis [62,63]. The analogy between cellular proliferation and metastasis formation in cancer and synovial hyperplasia and invasion in rheumatoid arthritis is well-recognized [64]. For that reason, and because tumor associated macrophages also contribute to cancer progression [4], we examined tumors from CD248^WT/WT ^and CD248^CyD/CyD ^mice for leukocyte infiltration (data not shown). There were, in fact, fewer leukocytes in the tumors from CD248^CyD/CyD ^mice (1000 + 28 cells/mm^2 ^in CD248^CyD/CyD ^*versus *1200 + 86 cells/mm^2 ^in CD248^WT/WT^, n = 7, p = 0.0578), although the differences were not statistically significant. Nonetheless, the findings suggest that the cytoplasmic domain of CD248 might also participate in the regulated release by activated fibroblasts of pro-inflammatory factors such as IL-1β, monocyte chemotactic protein (MCP)-1 and IL-8 [7], thereby further tipping the balance of the stromal microenvironment toward one that favours tumor initiation and progression.

A striking observation made by investigators who previously evaluated CD248-deficient mice in tumor models was the smaller tumor size associated with a seemingly paradoxical increase in microvessel density [21,25]. In spite of their finding that pericyte coverage was not altered, Nanda et al. postulated that CD248-deficient blood vessels may fail to mature properly, hence favoring the sprouting of small-caliber vessels [25]. Surprisingly, we did not observe any differences in vessel density or size distribution, nor in pericyte coverage in T241 fibrosarcoma tumors from the CD248^CyD/CyD ^mice or CD248^KO/KO ^mice. Several factors could explain why the angiogenic responses were different in our studies *versus *others, particularly for the CD248-deficient mice. First and foremost, the xenograft tumor cell lines that we examined were different from those of Nanda et al. [25] and Carson-Walter et al. [21]. Second, one of the groups [21] used immunodeficient mice for their xenograft studies. Finally, the mice were exposed to different environmental factors and were generated from distinct genetic backgrounds. In spite of these differences, the remarkable finding in the CD248^CyD/CyD ^mice and the CD248^KO/KO ^mice (irrespective of the source) was that tumor angiogenesis was not reduced, in spite of the tumors being smaller than in their wild-type counterparts. This paradoxical lack of reduced tumor angiogenesis in the setting of smaller tumors is not without precedent. For instance, Gas6-deficient mice grew smaller tumors as compared to their wild type counterparts, while microvessel density, vessel lumen area and pericyte coverage did not change [37]. In that case, it was determined that tumors cells induce infiltrating leukocytes to produce the mitogen Gas6. Since CD248 is not expressed by tumor cells, but rather by activated fibroblasts, it is interesting to note that conditioned media from CD248^CyD/CyD ^fibroblasts dampened the proliferative potential of T241 fibrosarcoma cells. Thus, it is likely that the cytoplasmic domain of CD248 facilitates fibroblast release of soluble factors that promote tumor growth. Several candidates could be considered, including for example, IGF-1, HGF and FGF, all of which favor tumor cell survival and proliferation [2-4,65].

Interestingly, we and others have shown that not all tumors depend on CD248 for growth. Moreover, the same tumor does not necessarily progress in the same CD248-dependent manner in different anatomical sites [25]. Several groups have demonstrated that tumor microenvironment and location functionally influence tumor growth and metastasis. And tumor stromal heterogeneity may be associated with multiple factors including differences in hypoxia-induced vascular response [66], vessel maturation [67] and activation of tumor associated fibroblasts [68]. Such heterogeneity has considerable clinical relevance and may explain, at least to some extent, unresponsiveness of some tumors to anti-VEGF therapy [69]. The variable contribution of CD248 to tumor growth highlights the importance of understanding and establishing multiple targets for the design of effective therapies for given tumors.

## Conclusion

Overall, our studies confirm that CD248 is an important central regulator of several critical pathways involved in stromal fibroblast migration, proliferation and activation that impact on tumor growth. Although we demonstrated the importance of the cytoplasmic domain of CD248, a structure that is highly conserved, with a PDZ binding motif and potential sites for phosphorylation, no intracellular interacting partners have yet been identified. Conversely, others have shown that the ectodomain of CD248 does interact with components of the ECM, including collagen, fibronectin and Mac-2 BP/90 K, and also participates in promoting tumor growth by modulating cellular adhesion and migration and promoting the release of MMP-9 [30,31]. It is likely that the intra- and extra-cellular domains of CD248 co-operate to achieve the same functional endpoint, but the mechanisms and responsible protein-protein interactions in the cell and outside the cell remain to be determined. By gaining further insights, one may ultimately consider therapeutic strategies to interfere with CD248 signaling pathways to "normalize", i.e. reverse the genotype/phenotype of the tumor associated fibroblast, thereby turning a tumor-permissive stromal environment into a tumor-prohibitive one.

## Competing interests

The authors declare that they have no competing interests.

## Authors' contributions

MM participated in the design of the project, coordinated and performed most of the experiments, and helped in the preparation of the manuscript. AV, TJ and MM provided technical support in managing mice, preparing cells, performing assays and analysing data. RJL and JT helped in the design of the studies and preparation of the manuscript. EMC conceived of the study and its design, supervised all aspects of the work, and prepared the manuscript. All authors read and approved the final manuscript.

## Pre-publication history

The pre-publication history for this paper can be accessed here:

http://www.biomedcentral.com/1471-2407/11/162/prepub
